# Recent advances in glucose-responsive insulin delivery systems: novel hydrogels and future applications

**DOI:** 10.1093/rb/rbac056

**Published:** 2022-08-23

**Authors:** Avha R Mohanty, Akhila Ravikumar, Nicholas A Peppas

**Affiliations:** McKetta Department of Chemical Engineering, The University of Texas at Austin, 200 E Dean Keeton St, Austin, TX 78712, USA; Institute for Biomaterials, Drug Delivery, and Regenerative Medicine, The University of Texas at Austin, 107 W Dean Keeton St, Austin, TX 78712, USA; Institute for Biomaterials, Drug Delivery, and Regenerative Medicine, The University of Texas at Austin, 107 W Dean Keeton St, Austin, TX 78712, USA; Department of Biomedical Engineering, The University of Texas at Austin, 107 W Dean Keeton St, Austin, TX 78712, USA; McKetta Department of Chemical Engineering, The University of Texas at Austin, 200 E Dean Keeton St, Austin, TX 78712, USA; Institute for Biomaterials, Drug Delivery, and Regenerative Medicine, The University of Texas at Austin, 107 W Dean Keeton St, Austin, TX 78712, USA; Department of Biomedical Engineering, The University of Texas at Austin, 107 W Dean Keeton St, Austin, TX 78712, USA; Division of Molecular Pharmaceutics and Drug Delivery, College of Pharmacy, The University of Texas at Austin, 2409 University Ave, Austin, TX 78712, USA; Department of Surgery and Perioperative Care, Dell Medical School, The University of Texas at Austin, 1501 Red River St, Austin, TX 78712, USA; Department of Pediatrics, Dell Medical School, The University of Texas at Austin, 1501 Red River St, Austin, TX 78712, USA

**Keywords:** insulin delivery, glucose control, hydrogels, glucose oxidase, diabetes

## Abstract

Over the past several decades, there have been major advancements in the field of glucose sensing and insulin delivery for the treatment of type I diabetes mellitus. The introduction of closed-loop insulin delivery systems that deliver insulin in response to specific levels of glucose in the blood has shifted significantly the research in this field. These systems consist of encapsulated glucose-sensitive components such as glucose oxidase or phenylboronic acid in hydrogels, microgels or nanoparticles. Since our previous evaluation of these systems in a contribution in 2004, new systems have been developed. Important improvements in key issues, such as consistent insulin delivery over an extended period of time have been addressed. In this contribution, we discuss recent advancements over the last 5 years and present persisting issues in these technologies that must be overcome in order for these systems to be applicable in patients.

## Graphical Abstract

**Figure:**
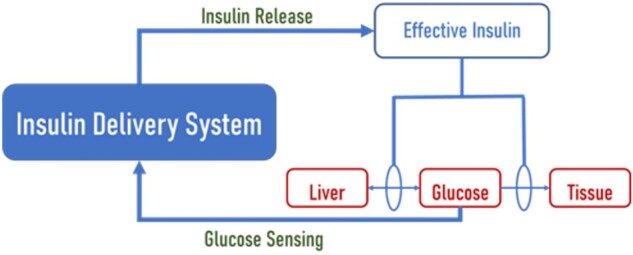

## Introduction

Diabetes mellitus is a disease characterized by the inability of the body to produce enough insulin or respond to insulin. This results in the accumulation of glucose in the blood, which can cause severe damage to essential organs. Diabetes can also lead to other serious health problems including cardiovascular disease, neuropathy, hypertension and stroke. This disease affects a growing number of people globally. According to the International Diabetes Foundation, the number of adults aged 20–79 suffering from diabetes is expected to increase by almost 50% from approximately 537 million in 2021–783 million in 2045 [1, 2].

Of the two types of diabetes, type I diabetes is an autoimmune disease that involves the destruction of beta cells in the pancreas, resulting in little to no insulin production. It typically appears in adolescence and is often referred to as juvenile-onset diabetes. This type of diabetes can only be treated with insulin therapy. Type II diabetes results in reduced insulin production or insulin resistance due to receptor desensitization. While genetics can play a role, a healthy diet and exercise can help prevent and control the disease.

Diabetes is a disease that must be continuously monitored and treated. Normal blood glucose levels are defined as below 140 mg/dl, while diabetic blood glucose levels are 200 mg/dl or higher. Prediabetes is characterized by blood glucose levels between 140 and 199 mg/dl [3]. Effectiveness of treatment options is essential to the quality of life of patients. Several technologies have been developed to monitor and treat the disease. The traditional method involves finger stick blood glucose monitoring paired with insulin delivery via subcutaneous injection [4]. This method consists of constantly pricking and injecting the patient which causes significant discomfort for the patient and may result in patient noncompliance. It also restricts the number of times a patient is able to monitor their glucose level and prevents them from seeing the full timeline of glucose levels throughout the day. To address this issue, continuous glucose monitoring (CGM) has been developed. With this method, a small enzymatic sensor with a needle attachment is inserted into the subcutaneous layer under the skin. It continuously monitors glucose levels and transmits data to an external device for the patient to view.

**Figure 1.**
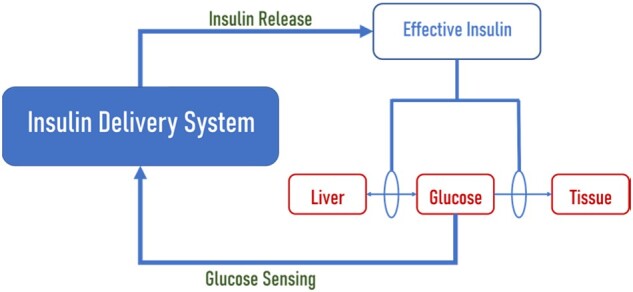
Schematic of closed-loop insulin delivery system in the body.

To consolidate the glucose sensing and insulin delivery involved in treating diabetes, several closed-loop systems have been developed. These systems, also known as *feedback-controlled devices,* eliminate the need for patient intervention. They are able to self-administer the insulin to the patient based on blood glucose data as shown in Fig. 1. Glucose oxidase (GOx) is often utilized in these systems to quantify the amount of glucose in the blood. In the presence of GOx, glucose reacts with oxygen to form gluconic acid and hydrogen peroxide, resulting in a lower pH that can be measured.

(1) $$\mathrm{Glucose}+O_{2}\overset{\mathrm{GOx}}{\to }\mathrm{Gluconic}\;\mathrm{acid}+H_{2}O_{2}.$$

Some examples of commonly used diabetes treatment systems are the Tandem/Dexcom Control-IQ and FreeStyle Libre [5, 6]. These systems combine an insulin delivery pump with a CGM device. The CGM system contains a small sensor containing a glucose-sensitive enzyme embedded in a needle that is inserted into the patient’s subcutaneous tissue. The device measures glucose levels, and the data are then transmitted to the insulin pump. The pump is an external device that contains an insulin reservoir that delivers insulin to the patient through an infusion set which injects insulin into the body. Typically, the patient replaces the sensors and pumps every 3–4 days [7]. While this method provides continuous data and eliminates the need for constant finger pricks, it still causes patient discomfort and requires frequent calibration.

**Figure 2.**
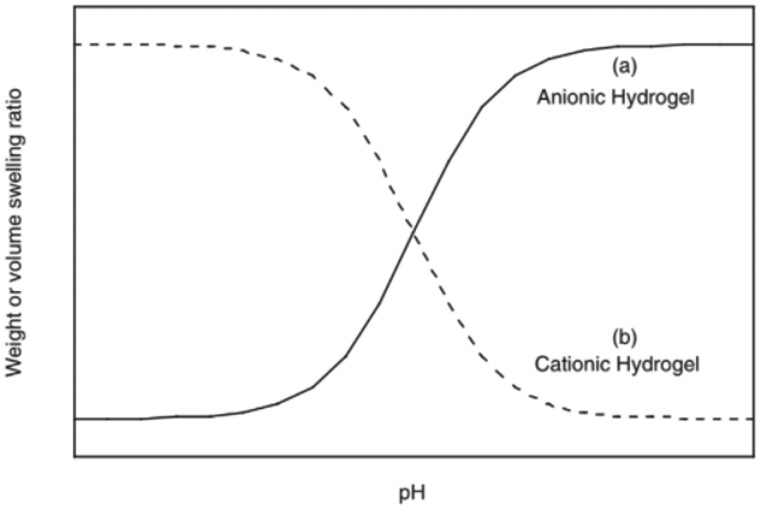
(a) Cationic hydrogels swell at higher pH whereas (b) anionic hydrogels swell at a lower pH. Figure reprinted from Peppas, 2004 with permission from Elsevier [8].

A method of closed-loop insulin delivery has been proposed and studied using hydrogels containing insulin, which assists in the delivery of therapeutic agents. These systems are distinct from traditional delivery systems as they deliver insulin upon stimulus by glucose rather than continuous and constant delivery. This glucose-dependent release allows the system to function as a valve, regulating the release of insulin from the main source of the drug within the carrier. The principal carriers of such devices are hydrogels, which are hydrophilic cross-linked polymers that can swell in water. One of the reasons that delivery from hydrogels is desirable is the fact that hydrogels show promise as biomaterials for a wide variety of applications including wound dressings, contact lenses and drug delivery. Specifically, ionic hydrogels can respond to various stimuli including pH. Based on the pK_a_ of the monomer components of the gel, anionic hydrogels will swell at higher pH levels, and cationic hydrogels will swell at lower pH levels as shown in Fig. 2.

For drug delivery applications, pH-sensitive swelling can be utilized to release an incorporated drug at a target pH. Thus, pH-sensitive hydrogel nanoparticles can be used for closed-loop insulin delivery. In this system, insulin, along with the enzymes GOx and catalase are encapsulated in a cationic hydrogel. As glucose levels rise, glucose reacts in the presence of GOx to form gluconic acid and hydrogen peroxide, lowering the pH. The cationic hydrogel then swells at the lower pH level, increasing the mesh size of the hydrogel. Thus, molecular pores are presented, and they allow insulin release. The addition of catalase allows hydrogen peroxide to further react to replenish the depleted oxygen. However, it is important to note that the catalase reaction can only regenerate half of the original oxygen content as half of the oxygen is incorporated into the gluconic acid.

Several hydrogel-based systems for feedback-regulated insulin delivery have been developed using a variety of materials, with varying crosslinking densities and incorporating enzymes. In previous work, we discussed some of the early efforts in this area [8]. Since then, there have been significant changes in our evaluation of these systems and in the types of biomaterials used for these systems. However, several key drawbacks have prevented these systems from progressing to clinical applications. A high quantity of insulin must be encapsulated in a small system to prevent re-administration of the system over a reasonable time interval. Additionally, as insulin is released and depleted from the system, the laws of diffusion prevent a consistent amount of insulin delivery over an extended period of time.

Optimal insulin release kinetics from hydrogel systems is also challenging to achieve in practice. In theory, a hydrogel should exhibit three distinct phases over the span of at least 3 weeks. An initial burst phase releases the largest amount of insulin on Day 1. This is followed by a decelerating release phase in which the insulin gradient between the hydrogel and the surrounding environment is reduced. Finally, a constant release phase should be observed once 70–75% of the time [9] since insulin administration is spent in euglycemia [10].

Hysteresis is the process whereby there is a delay from expansion to contraction or very often there is a significant change in the speed of delivery of insulin from these systems. Ideally, we would like to be able to have the same amount delivered each time. Because the concentration gradient of insulin may change from the first to the fifth or the tenth application, it is conceivably difficult to see how one can develop a system that will deliver insulin at a constant rate for a long period of time.

These systems must also meet certain biocompatibility standards based on the type and location of the device within the body. For devices residing in the subcutaneous tissue, moisture content and foreign body response are especially important. Conversely, for systems that circulate the blood, thromboresistance and the use of stealth agents such as poly(ethylene glycol) (PEG) are more important. All devices should meet FDA standards for cytotoxicity and biocompatibility [11]. It must also be noted that the systems can be prepared in the presence of a biodegradable cross-linking agent that can eventually be eliminated from the kidney. Thus far, systems have been designed such that the final degradable material has a molecular weight <20 000 kDa, which can be cleared by the kidney.

The stability of the protein is yet another important consideration in designing a protein delivery system. Within the body, most insulin degradation occurs intracellularly. However, some extracellular insulin degradation can occur in the presence of insulin degradation enzyme (IDE). [12] The stability of insulin can be analyzed using reverse-phase high-performance liquid chromatography (RP-HPLC) with ELISA after release from the system to ensure it does not degrade readily [13].

Given the current drawbacks that have persisted for many years, there are significant challenges that limit the progress of this technology. In this contribution, we address current technologies for closed-loop insulin delivery and highlight key issues with these systems.

## Hydrogels as sensors

### pH-sensitive hydrogels as carriers

The suitability of a hydrogel as a drug delivery device and its performance in a particular application depends to a large extent on its bulk structure. The most important parameters used to characterize the network structure of hydrogels are the polymer volume fraction in the swollen state, the molecular weight of the polymer chain between two neighboring crosslinking points and the corresponding mesh size. The polymer volume fraction in the swollen state (i.e. the percent by volume of the swollen polymer that is dry polymer) is a measure of the amount of fluid imbibed and retained by the hydrogel. The molecular weight between two consecutive cross-links, tie-junctions or physical entanglements which can be either of chemical or physical nature, is a measure of the degree of crosslinking of the polymer. It is important to note that due to the random nature of the polymerization process itself only average values of the polymer molecular weight between crosslinks can be calculated. The correlation distance between two adjacent cross-links provides a measure of the space available between the macromolecular chains available for the drug diffusion; again, it can be reported only as an average value. These parameters, which are related to one another, can be determined theoretically or through the use of a variety of experimental techniques. Two methods that are prominent among the growing number of techniques utilized to elucidate the structure of hydrogels due to their frequent use are the equilibrium swelling theory and the rubber elasticity theory [14].

pH-sensitive hydrogels are designed to swell or collapse depending on the pH of their surrounding environment. There are two primary mechanisms that can cause this swelling behavior. The first involves a transition away from hydrophobic interactions in favor of hydrophilic interactions. This mechanism relies on the presence of ionized side chains that increase the net hydrophilicity of the polymer network and cause water to swell into the matrix. The second mechanism involves the disruption of the hydrogen bonds that hold complexes together. This mechanism suggests that the ionization of side chains interferes with hydrogen bonds and unravels complexes in order to provide a pathway for water entry into the matrix. Regardless of the mechanism used, the swelling profile of hydrogels is also shaped by other factors like the ionic strength of the medium, buffer composition and concentration of salts.

**Figure 3.**
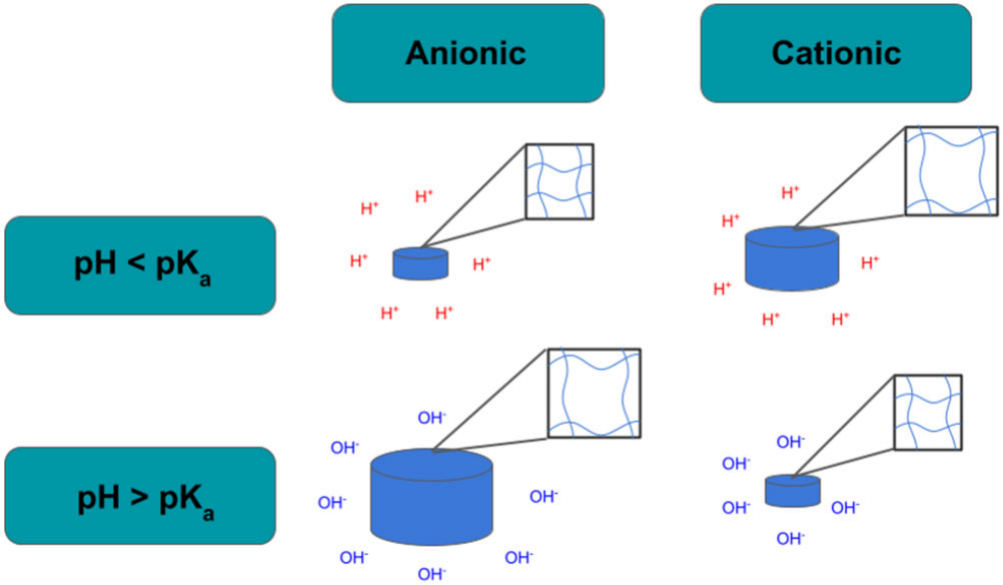
An overview of how anionic and cationic polymers respond to different pH conditions. Anionic hydrogels collapse at a low pH and swell at a high pH, while cationic hydrogels display the opposite behavior and collapse at a high pH and swell in a more acidic environment.

pH-responsive hydrogels typically fall into one of two classes: anionic or cationic. An overview of the swelling behavior of these hydrogels is shown in Fig. 3. Anionic hydrogels tend to have a negatively charged group like a carboxylic acid (COOH). At low pH values, anionic hydrogels are in the collapsed state because the pH is below the pKa of the acid groups. Once the pH rises above the pKa, the carboxylic acid is deprotonated (COO^−^), and there is a build-up of negatively charged groups that experience strong electrostatic repulsion with each other. Ultimately, it is the close proximity between similarly charged chains that changes the conformation of the hydrogel and enables it to absorb water [8].

Some common anionic polymer materials include poly(methacrylic acid) and poly(acrylic acid). These acrylic acid derivatives are notable for their strong adhesiveness and pH dependence [15]. These properties are harnessed in intestinal drug delivery applications in particular because they stabilize the drug while in the hydrogel and prevent early release of the drug from the hydrogel [16]. Cationic hydrogels on the other hand carry a positively charged group such as an amine (NH_2_) in their polymer backbone. When the pH is higher than the pKa of the cationic group, the hydrogel exists in a collapsed state because hydrophobic interactions dominate and prevent water from flowing into the matrix. However, once the pH is less than the pKa, the amine is protonated (${\text{NH}}_{3}^{+}$), and the matrix becomes more hydrophilic in nature, prompting the hydrogel to swell.

Examples of cationic polymers found in hydrogels include chitosan, polyethylenimine (PEI) and poly(L-lysine) [17]. Hariharan and Peppas [18] studied the suitability of cationic hydrogels composed of diethylaminoethyl methacrylate (DEAEM) and 2-(diethylamino)ethyl acrylate (DEAEA) copolymerized with poly 2-(hydroxyethyl)methacrylate (HEMA) for drug delivery applications. The swelling studies revealed that the hydrogel system displayed a gradual transition from the collapsed to the swollen state. The low concentration of ionized groups in the polymer chain minimized electrostatic repulsion and thereby contributed to a slower rate of water absorption in the hydrogel. However, a gradual transition is desirable because it enables the hydrogel to absorb more water compared to a sharp transition between the collapsed and swollen states. This prevents the possibility of an abrupt cutoff in insulin [19]. Moreover, the studies demonstrated that the ionic strength and pH of the external medium affected the quantity and rate of water absorbed into the hydrogel matrix; an increase in ionic strength was coupled with a decrease in water absorption while a decrease in pH was paired with an increase in the rate of water absorption [8, 18].

### Glucose-sensitive hydrogels

Glucose-sensitive hydrogels are viable carriers for insulin delivery because they can provide a biphasic release profile based on the glucose levels present in the bloodstream. For example, high glucose concentrations (hyperglycemia) necessitate quick insulin delivery, and these hydrogels can release insulin when needed without requiring any patient input. Conversely, during low blood glucose conditions (hypoglycemia), insulin is not required, and the hydrogels may control and stop insulin delivery. Therefore, glucose-sensitive hydrogels can serve as a potential self-monitoring device for diabetics. The key element behind these hydrogels is a glucose sensor that can detect glucose concentration and its rate of change in order to diagnose whether insulin is needed or not. The three major glucose sensors employed in these hydrogels are Concanavalin A (Con A), phenylboronic acid (PBA) and GOx.

The most common enzyme in glucose sensors is GOx. The success of this enzyme in biosensor applications is largely due to its high specificity, stability and turnover [20]. GOx converts glucose and oxygen to gluconic acid, with a particularly useful reaction that decreases the environmental pH. This change can be measured quantitatively and utilized for glucose sensing. Importantly, GOx is stable at a physiological pH range. It is most stable at a pH of 5 and begins to degrade below a pH of 2 and above a pH of 8 [20]. This range makes it a suitable enzyme for glucose sensing.

Further, lyophilized GOx has an extremely long shelf life ranging from 2 to 8 years depending on the storage temperature, facilitating storage and usage of the enzyme in many glucose sensing devices. However, GOx is slightly limited by its low molecular weight, which decreases the efficiency of its turnover number relative to other enzymes used in glucose sensing such as quinoprotein glucose dehydrogenase [20]. Further, enzyme-based sensors are limited by their inability to directly measure glucose concentrations. Only the environmental changes caused by the newly formed products of the induced reaction are measured. Still, GOx remains the most widely used enzyme for glucose sensing applications.

An early example of a GOx containing closed-loop insulin delivery system was developed by Ishihara and collaborators [21]. The system consisted of GOx immobilized in an amphiphilic polyamine membrane *N*,*N*-diethylaminoethyl methacrylate (DEA) and 2-hydroxypropyl methacrylate copolymer. Due to the formation of gluconic acid from glucose in the presence of GOx, the resulting decrease in pH would cause protonation of tertiary amine groups in the polyamine membrane. This induces a structural change that increases the permeability of the membrane, allowing the diffusion of insulin through the membrane. Later developments in insulin delivery would utilize these principles to produce unique variations of hydrogels as sensors.

Another example of a GOx system was developed by Podual *et al.* [22] who created a glucose-sensitive insulin delivery system in the form of pH-sensitive hydrogel nanoparticles. The nanoparticles were comprised of poly(diethylaminoethyl methacrylate-g-ethylene glycol) P(DEAEM-g-EG) and contained insulin, GOx and catalase. As GOx converts glucose to gluconic acid, decreasing pH, the cationic hydrogel would swell, allowing the release of insulin from within the particle. Additionally, catalase in the particles converted hydrogen peroxide from the primary reaction into oxygen, preventing oxygen depletion in the system and improving the overall effectiveness of the particles. This system effectively released insulin in a glucose-dependent fashion and showed promise for the future of closed-loop insulin delivery.

PBAs have been proposed as an alternative to GOx for glucose sensing applications. They are particularly effective due to their ability to bind to glucose in a fully reversible process, making it suitable for continuous measurements. PBA acts as a covalent receptor for cis-1,2- or -1,3-diols of glucose to form five- or six-membered rings groups of glucose molecules. These ring structures affect light absorbance which can be measured to quantify glucose levels. This is favorable for optical glucose sensing utilizing techniques such as surface plasmon resonance, surface-enhanced Raman scattering and photonic band gap sensors to measure changes in light absorbance due to changing glucose levels [22]. Importantly, this process does not consume any glucose or form any products that may interfere with the longevity of the sensor. Therefore, this eliminates the need for constant recalibration and enables long-term glucose monitoring. Several wearable continuous glucose monitors have used PBA-containing fluorophores. However, fluorescence-based sensing is susceptible to photobleaching and interference from external light sources.

Other methods of optical sensing using PBA have been developed to overcome these challenges. Worsley *et al*. immobilize PBA in a hydrogel containing holographic grating. In this system, glucose binds to PBA, causing the hydrogel to swell. This increases the spaces between the holographic fringes of the grating and thus changes the wavelength of diffracted light which can be measured and used to calculate glucose levels [23]. Disadvantages of PBA include its nonspecific nature. Because PBA can bind to cis-diols, it can bind to several different carbohydrates, including sialic acid, galactose, mannose and fructose. The competitive binding of coexisting carbohydrates in the blood and in glycoproteins to PBA at low glucose concentrations could interfere with such systems [24]. PBA is also limited by its high pK_a_ value. With a pK_a_ value greater than 8, PBA does not function at physiological pH. Thus, many studies have focused on formulating PBA-based polymers to effectively decrease the pK_a_ of PBA moiety to allow insulin release at physiological pH [25]. Another concern is the safety risk of PBA into the blood due to highly pH-dependent acid–diol interactions [23]. Though PBA shows promise as an alternative to enzyme-based sensors, further studies are required to overcome these limitations.

Another less common compound that has been used for glucose sensing is Concavalin A (ConA). ConA is a lectin extracted from the jack bean (*Canavalia ensiformis*) that binds specifically to glucose and can be utilized in optical glucose sensors with a similar mechanism to PBA. Several research groups have successfully tested ConA glucose sensors that remain functional for up to 6–12 months [26]. Despite these impressive results, ConA has been found to be associated with several biological defects including mitogenesis, hepatotoxicity and teratogenicity [26]. Though usage of this compound in small quantities has shown little to no harmful effects *in vivo*, the potential risks do not make ConA the most attractive option for glucose sensing.

### Redox-responsive hydrogels

Redox-responsive hydrogels swell due to oxidation–reduction reactions. There are notably few systems that choose to use oxidation-responsive hydrogels perhaps due to their low sensitivity, slow response rate to reactive oxygen species (ROS), or simply because they lack the mechanical integrity that can be found in other hydrogel systems. Despite these shortcomings, redox-responsive hydrogels are biocompatible and contain oxidation-sensitive motifs that are highly sensitive to compounds like hydrogen peroxide that make them suitable candidates for insulin drug delivery. Zhang *et al.* [27] explored this potential and developed a redox-responsive PEG hydrogel system with a hydrogen peroxide cleavable PBA linker. This system was designed to deliver insulin by sensing the ROS hydrogen peroxide, which is a byproduct that forms after GOD converts glucose into gluconic acid.

In 2012, Ishihara *et al.* [28] further explored the ability of GOx to react continuously in redox reactions. They immobilized GOx in redox phospholipid polymer microparticles and measured electron transport to a gold electrode. They found that the immobilization of enzymes onto solid particles allows for continuous enzymatic reaction and electron transfer. These findings have been used to further develop biosensors like GOx in closed-loop insulin delivery devices.

## Recent advances in insulin-responsive hydrogel systems

### GOx-based systems

**Figure 4.**
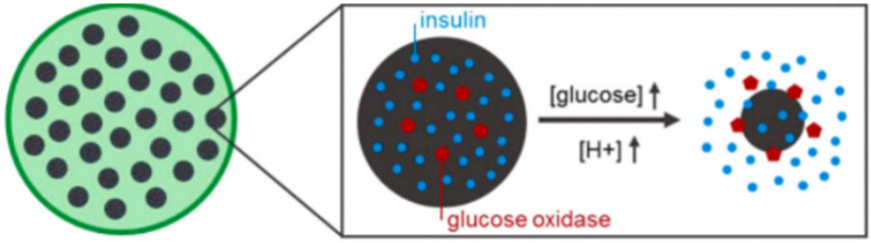
Glucose-responsive insulin release from Ac-dextran nanoparticles encapsulated in alginate microgels. Figure adapted from Volpatti *et al.* [29] with permission from Elsevier.

In the last 5 years, there have been numerous efforts to utilize hydrogels in the form of thin films, spheres or cylindrical devices and incorporate them in a device that will be able to detect high concentrations of glucose in the presence of one or more enzymes and lead to prolonged delivery of insulin either directly in the blood or in tissue. For example, Langer and collaborators developed a system consisting of encapsulated glucose-responsive, acetylated-dextran nanoparticles in porous alginate microgels containing GOx and insulin [29]. Under acidic conditions produced by the formation of gluconic acid from glucose in the presence of GOx, acetal bonds in the polymer are cleaved, allowing nanoparticles, and thus insulin to be released as depicted in Fig. 4.

Studies were performed both *in vitro* and *in vivo* in mice to quantify insulin release and blood glucose levels over time. Results of the studies revealed that the device provided insulin release for 12 h *in vitro* and controlled blood glucose levels for approximately 10 days *in vivo*, with the highest insulin release after 4 h of incubation.

Additionally, fluorescence imaging was conducted *in vivo* to observe microgels and free nanoparticles loaded with insulin conjugated with fluorescein isothiocyanate (FITC) under hyperglycemic conditions (glucose concentration of 400 mg/dl) [29]. Results supported that microgel encapsulation of the nanoparticles improved the stability of the device by preventing movement of the nanoparticles from the initial site of injection, preventing premature degradation and allowing larger doses of nanoparticles. Additionally, the presence of a microgel prevented leakage of insulin from the system. Because insulin is a relatively small molecule with a size less than 6 kDa, previous systems showed leakage of insulin molecules through hydrogel pores which typically had pore sizes larger than the size of insulin at physiological pH. While this system showed promising results, further systems were developed to improve the biocompatibility of closed-loop insulin delivery.

**Figure 5.**
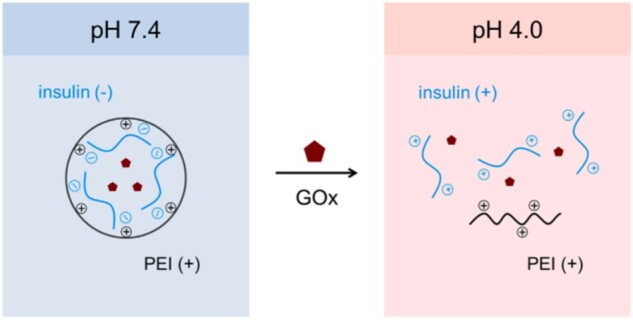
Glucose-responsive insulin release from disassembly of electrostatic complexes. Figure reprinted from Volpatti *et al.* [30] with permission from Elsevier Science Ltd.

Langer and collaborators also explored a new method of closed-loop insulin delivery by developing electrostatic complexes (ECs) containing a polycation, GOx and insulin. Unlike the previous system, this device relies on disruptions in charge interactions to dissemble the EC’s and release insulin. When blood glucose levels are within the normal limits, insulin molecules are negatively charged and form stable EC’s with the polycation. When glucose levels rise, GOx converts glucose into gluconic acid. This lowers the pH and results in a positive charge on the insulin. The disruption of charge attraction between the polycation and insulin, resulting in the disassembly of EC’s and thus, the release of insulin as seen in Fig. 5.

EC’s were synthesized via double emulsion solvent evaporation resulting in insulin loading efficiencies between 58 and 66%. Molecular dynamics simulations were conducted to model charge interactions and determine *in vitro* release kinetics. Release profiles showed glucose-dependent release of insulin for 6–18 h that also corresponded to measured changes in pH. Approximately half of the loaded insulin was released in the first 2 h of release testing [30]. EC’s offer solutions to the issues posed by the previously mentioned nanoparticle insulin delivery system. Due to the continuous degradation of the system, EC’s are more applicable to real-life applications of the system in the human body as they allow for repeated dosing without the accumulation of materials at the site of injection. However, continuous delivery of the same quantities of insulin over an extended period of time remains a challenge.

### PBA-based insulin delivery systems

PBA has been investigated as an alternative glucose sensor to GOx in novel insulin delivery systems. Notably, PBA can reversibly bind to 1,2- or 1,3-cis diols like glucose and is promising for accurate glucose sensing and sustained insulin delivery [31]. PBA derivatives are also more receptive to chemical modification compared to their counterparts GOx and ConA in order to achieve desired glucose sensitivity [32]. Recent studies have optimized these characteristics of PBA by embedding it in their insulin delivery systems.

Zhang *et al*. [27] developed redox-responsive 4-arm-PEG hydrogels with hydrogen peroxide cleavable PBA linkers via a radical polymerization reaction. These hydrogels can encapsulate insulin or GOx at high loading efficiencies of 95% and 96%, respectively. The release of the insulin or GOx inside the hydrogels is initiated by the presence of glucose or hydrogen peroxide in the surroundings. PBA is incorporated into the covalent polyethylene (PEG) hydrogel network to enable the rapid release of insulin even when hydrogen peroxide concentrations are low.

Insulin delivery from macroscopic hydrogels and nanogels was studied over a 12-h period, and oxidative degradation was analyzed. Results from the study demonstrate that adjusting the concentrations of glucose, hydrogen peroxide or GOx affects the insulin release profile. For example, 5.6 mM of hydrogen peroxide triggered the complete degradation of the hydrogel and subsequent release of insulin within 5 h. A low concentration of GOx (∼0.001 wt%) was observed to generate modest insulin release under hyperglycemic conditions, so the hydrogels can be used for *in vivo* applications. Zhang *et al.* also discovered that the GOx and the hydrogel experienced diverging degradation mechanisms; hydrogen peroxide caused bulk degradation by disrupting crosslinks, while glucose-initiated surface degradation of the hydrogel. This is the first known case of oxidative-responsive hydrogels using two separate degradation techniques from two different stimuli. Despite the significance of this finding, the study is subject to limitations; the *in vitro* results reveal that some of the GOx became inactive during radical polymerization suggesting that the oxidation of glucose was not entirely efficient and can be improved. Additionally, higher rates of insulin release were correlated with the rapid degradation of the hydrogel, so the system was not stable for long [27].

Another group of researchers, Lee *et al.* [33], designed a trehalose-boronic acid hydrogel system for controlled insulin delivery. The hydrogels were fabricated with trehalose polymers and PBA end-functionalized PEG. During hydrogel formation, the hydroxyl groups on the trehalose polymer chains form ester linkages with the PBA functionalized PEG. When glucose is present, it uses its diols to bind to PBA and displace the diols of trehalose causing the hydrogel to dissolve and insulin to be released. The cleavage of ester bonds further contributes to the release of insulin from the hydrogel at a neutral pH. Lee *et al.* experimentally derived binding affinity values to determine whether glucose can competitively displace trehalose.

Their findings validate that glucose binds more strongly to boronic acid compared to trehalose given its binding affinity (2.57 M^−1^) was nearly 5.4 times that of trehalose (0.48 M^−1^). Lee *et al.* also measured the quantity of glucose needed for hydrogel dissolution. Hydrogels placed in concentrated solutions of 1000 and 2000 mg/dl dissolved completely within 10 min, while hydrogels in less concentrated glucose solutions of 100 and 500 mg/dl reformed after an hour. FITC insulin release studies were performed with three different glucose concentrations (0, 500 and 1000 mg/dl) over the span of 2 h. An accelerated insulin release profile was seen at high glucose concentrations while slower insulin release was detected at lower glucose concentrations. Insulin release was also observed to be slower at a basic pH compared to a physiological pH, suggesting that the pKa of the boronic acid can be fine-tuned depending on the release profile required. A heating assay was performed to assess how well the trehalose hydrogel preserved insulin at an elevated temperature of 90°C for 30 min. Interestingly, the ELISA results confirm that the trehalose hydrogel had a stabilizing effect on the insulin despite the heat and loss of water experienced under these conditions [33].

Liu *et al.* [34] devised a polymeric micelle insulin delivery system that responds to both glucose and hydrogen peroxide. The system was formed from the block copolymer poly(ethylene glycol)-block-poly(amino phenylboronic ester) (PEG-b-PAPBE) via a Michael addition polymerization and is sensitive to hydrogen peroxide. The micelles were synthesized by adding the surfactant tetrahydrofuran dropwise into the block copolymer. Although the micelles were inherently glucose responsive, GOx was encapsulated inside them to further strengthen their glucose sensitivity. At high glucose concentrations, the PAPBE in the micelles reacts with glucose leading the PBE to break off and a new PBA-glucose complex to form.

**Figure 6.**
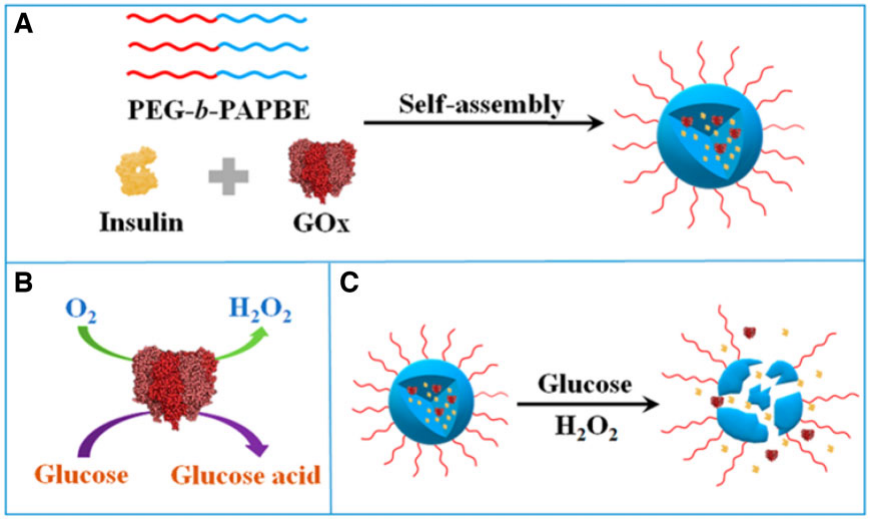
(**A**) Self-assembly of polymeric micelles. (**B**) Glucose oxidation reaction. (C) Glucose-responsive insulin release from polymeric micelles. Figure adapted from Liu *et al.* [34].

Simultaneously, GOx catalyzes the conversion of glucose into gluconic acid and produces hydrogen peroxide as a byproduct, which also hydrolyzes PAPBE. This gives the polymeric micelles their dual-responsive nature; they can respond to both glucose and hydrogen peroxide to initiate insulin delivery as shown in Fig. 6.

Dynamic light scattering testing was performed on the polymer micelles to gauge the extent of glucose and hydrogen peroxide responsiveness. The micelles’ response to glucose was satisfactory, while the response to hydrogen peroxide was excellent. Controlled release studies of the insulin over a 30-h period revealed that the addition of GOx to the micelles led to a faster insulin release rate.

This validated the assumption that GOx enhances the glucose-responsiveness of the polymer micelles. *In vivo* testing conducted on type I diabetic mice induced by STZ demonstrated that the polymer micelles were able to lower blood glucose levels without the risk of hypoglycemia. A hematoxylin and eosin staining showed that the micelles were biocompatible *in vivo* and did not trigger an inflammatory response to the tissue or organ damage in the diabetic mice. However, despite the therapeutic effectiveness of this system in the diabetic mice, it remains to be tested on other animal models for an extended period of time [34].

Another less common method for closed-loop therapeutic delivery that has been studied recently is transdermal delivery. Zhang *et al.* [35], developed a hydrogel system including arginine-based polyester amide (Arg-PEA) and polyethylene glycol diacrylamide (PEG-DA) along with insulin and transdermal peptide (TD-1), which is a short synthetic peptide that creates a transient opening in the skin to allow proteins to reach systemic circulation. The hydrogels are synthesized via UV photopolymerization and attach externally to the skin as a form of non-invasive, self-administered insulin therapy that can avoid hepatic metabolism.

Several tests were performed to measure gel degradation, skin biocompatibility, insulin release *in vitro* and blood glucose levels *in vivo*. Results showed that hydrogels did not interfere with normal water evaporation rates from the skin. The hydrogels would typically degrade after 35 days. *In vitro* insulin release rates showed the largest increase in the first 9 h and then slowed down after 12. *In vivo* studies of blood glucose levels in streptozotocin (STZ)-induced diabetic mice showed that TD-1 was essential for insulin delivery as it resulted in lower blood glucose levels. Baseline glucose levels were observed after 12 h of treatment [35]. While this system offers a non-invasive alternative to insulin delivery, external hydrogels may be more susceptible to environmental factors that could affect the moisture content and swelling properties of the hydrogel. Additionally, transdermal insulin delivery appears to deliver less insulin than other methods described.

Table 1 summarizes hydrogel-based glucose-responsive insulin delivery systems over the last 5 years, including hydrogel components, response mechanisms and important outcomes.

**Table 1.** Insulin delivery system studies for type 1 diabetes

Insulin delivery system	Glucose sensor	Hydrogel components	Key outcome	References
Microgel encapsulated nanoparticles	GOx	Alginate microgels	Microgel encapsulation of nanoparticles stabilized the system and deterred insulin leakage	[29]
		Ac-Dex nanoparticles		
		Catalase		
		Insulin		
Engineered insulin-polycation complexes	GOx	PEI electrostatic complex	EC’s allow for repeated dosing without excessive buildup at the injection site	[30]
		Insulin		
Oxidation-responsive biodegradable PEG hydrogels	PBA	4-arm-PEG	Glucose caused surface degradation of the hydrogel, while H_2_O_2_ triggered bulk degradation	[27]
		Insulin		
Trehalose hydrogel	PBA	Poly(SET)	Trehalose hydrogel stabilized the insulin	[33]
		8-arm-PEG boronic acid		
		Insulin		
Glucose and H_2_O_2_ dual responsive polymeric micelles	PBA	PEG-b-PAPBE	Micelles’ sensitivity to glucose was satisfactory, while the sensitivity to hydrogen peroxide was excellent	[34]
		Insulin		
Poly(ester amide) transdermal hydrogels	PBA	Arg-PEA	TD-1 was required to deliver insulin and lower blood glucose levels	[35]
		PEG-DA		
		TD-1		
		Insulin		

## Conclusions

Diabetes is a condition marked by the insufficient or ineffective use of insulin. It triggers erratic changes in glucose levels that need to be monitored and regulated by diabetics in order to prevent serious health complications like heart disease or kidney damage. Diabetes is demanding in nature because it requires the patient to repeatedly inject themselves with insulin multiple times a day. Interest in creating a less invasive device that can relieve patients from the responsibility of self-administering insulin has grown.

Such a device would enable patients to take a more hands-off approach in their diabetes management and simplify their day-to-day insulin regimens. In recent years, smart hydrogels have emerged as favorable biomaterials for use in physiological systems because they are biocompatible, biodegradable and responsive to external stimuli like pH, temperature and ionic strength. In this paper, an overview of current intelligent hydrogel-based insulin delivery systems was presented and discussed. Some systems utilize enzyme-dependent glucose detection like GOx, while others use non-enzymatic PBA-based mechanisms to trigger insulin release. GOx-based systems rely on a local pH change to promote insulin delivery. PBA-mediated systems utilize PBA’s cis-diol binding ability to sense glucose and modulate insulin release. However, despite extensive studies on hydrogels as insulin carriers being published in the last 20 years, there are no FDA-approved hydrogel devices available for insulin delivery. A few critical barriers exist that can explain why this is the case. One obvious concern is that the longevity of hydrogel-based insulin release systems cannot sustain the insulin demands needed for clinical use. Current release systems are only able to release insulin once or twice before needing to be replenished. Successful devices should be able to release insulin at least ten times before re-administration is necessary. Another issue is that hydrogel-based insulin delivery systems need to swell and shrink without the presence of hysteresis. Hysteresis enables a variable change in the speed of insulin delivery that makes consistent insulin delivery a challenge. Hysteresis may be reduced by using softer hydrogel materials or by integrating polyprotein crosslinkers. The risk of hypoglycemia is also high when delivering the initial dose of insulin. To minimize the likelihood of either hypoglycemia or hyperglycemia, a delivery system dually loaded with insulin and glucagon can balance deviating glucose levels. Furthermore, the safety of the patient cannot be compromised, so biocompatibility and the likelihood of a thrombotic event in the blood need to be assessed. Sustained long-term biocompatibility *in vivo* should be established with minimal to no toxicity.

Given the challenges ahead, further research is needed in the design of hydrogel systems, so that their longevity and diffusion capabilities are optimized. Therefore, these refined systems will be a promising therapy for diabetics because they will deliver insulin on-demand and help diabetics manage their blood sugar effectively in the long term.

## Funding

This work was supported in part by the Dean of the Cockrell School of Engineering at The University of Texas at Austin for the Institute for Biomaterials, Drug Delivery and Regenerative Medicine; and the UT-Portugal Collaborative Research Program.

*Conflicts of interest statement*. None declared.

## References

[1] International Diabetes Federation. Diabetes facts & figures. 2021.

[2] American Diabetes Association. Diabetes overview. 2022.

[3] Centers for Disease Control and Prevention. Diabetes. 2021.

[4] Olansky L , Kennedy L. Finger-stick glucose monitoring: issues of accuracy and specificity. Diabetes Care 2010;33:948–9. 20351231 10.2337/dc10-0077 PMC2845057

[5] Care TD. Control IQ technology. 2022.

[6] Abbott. FreeStyle Libre. 2022.

[7] Mansoor S , Kondiah P, Choonara Y. Advanced hydrogels for the controlled delivery of insulin. *Pharmaceuticals* 2021;13:2113. 10.3390/pharmaceutics13122113 PMC8703368 34959394

[8] Peppas NA. Is there a future in glucose-sensitive, responsive insulin delivery systems? J Drug Deliv Sci Technol 2004;14:247–56.

[9] Senior P , Lam A, Farnsworth K, Perkins B, Rabasa-Lhoret R. Assessment of risks and benefits of beta cell replacement versus automated insulin delivery systems for type 1 diabetes. Curr Diab Rep 2020;20:52. 32865637 10.1007/s11892-020-01339-3

[10] Zhao F , Wu D, Yao D, Guo R, Wang W, Dong A, Kong D, Zhang J. An injectable particle-hydrogel hybrid system for glucose-regulatory insulin delivery. Acta Biomater 2017;64:334–45. 28974477 10.1016/j.actbio.2017.09.044

[11] Use of International Standard ISO 10993-1, “Biological evaluation of medical devices - Part 1: Evaluation and testing within a risk management process”. Administration USFaD. 2020:30–45.

[12] Duckworth W , Bennett R, Hamel F. Insulin degradation: progress and potential. Endocrine Rev 1998;19:608–24. 9793760 10.1210/edrv.19.5.0349

[13] Kim B , Peppas N. In vitro release behavior and stability of insulin in complexation hydrogels as oral drug delivery carriers. Int J Pharm 2003;266:29–37. 14559391 10.1016/s0378-5173(03)00378-8

[14] Peppas NA , Bures P, Leobandung W, Ichikawa H. Hydrogels in pharmaceutical formulations. Eur J Pharm Biopharm 2000;50:27–46. 10840191 10.1016/s0939-6411(00)00090-4

[15] Bashir S , Hina M, Iqbal J, Rajpar A, Mujtaba M, Alghamdi N, Wageh S, Ramesh K, Ramesh S. Fundamental concepts of hydrogels: synthesis, properties, and their applications. Polymers 2020;12:2702. 10.3390/polym12112702 PMC7697203 33207715

[16] Bilia A , Carelli V, Di Colo G, Nannipieri E. In vitro evaluation of a pH-sensitive hydrogel for control of GI drug delivery from silicone-based matrices. Int J Pharm 1996;130:83–92.

[17] Tabujew I , Peneva K. Chapter 1: Functionalization of cationic polymers for drug delivery applications. In: Cationic Polymers in Regenerative Medicine. The Royal Society of Chemistry 2015,1-29.

[18] Hariharan D , Peppas NA. Characterization, dynamic swelling behaviour and solute transport in cationic networks with applications to the development of swelling-controlled release systems. Polymer 1996;37:149–61.

[19] Ahmed E. Hydrogel: preparation, characterization, and applications: a review. J Adv Res 2015;6:105–21. 25750745 10.1016/j.jare.2013.07.006 PMC4348459

[20] Wilson R , Turner APF. Glucose oxidase: an ideal enzyme. Biosensors Bioelectron 1992;7:165–85.

[21] Ishihara K , Kobayashi M, Ishimaru N, Shinohara I. Glucose induced permeation control of insulin through a complex membrane consisting of immobilized glucose oxidase and a poly (amine). Polym J 1984;16:625–31.

[22] Elsherif M , Hassan MU, Yetisen AK, Butt H. Glucose sensing with phenylboronic acid functionalized hydrogel-based optical diffusers. ACS Nano 2018;12:2283–91. 29529366 10.1021/acsnano.7b07082 PMC5916466

[23] Worsley GJ , Tourniaire GA, Medlock KES, Sartain FK, Harmer HE, Thatcher M, Horgan AM, Pritchard J. Measurement of glucose in blood with a phenylboronic acid optical sensor. J Diabetes Sci Technol 2008;2:213–20. 19885345 10.1177/193229680800200207 PMC2771504

[24] Smoum R , Srebnik M. Contemporary aspects of boron: chemistry and biological applications. Stud Inorgan Chem 2005;22:391–494.

[25] Lan T , Guo Q. Phenylboronic acid-decorated polymeric nanomaterials for advanced bio-application. Nanotechnol Rev 2019;8:548–61.

[26] Ballerstadt R , Evans C, McNichols R, Gowda A. Concanavalin a for in vivo glucose sensing: a biotoxicity review. Biosensors Bioelectron 2006;22:275–84. 10.1016/j.bios.2006.01.008 16488598

[27] Zhang M , Song C-C, Du F-S, Li Z-C. Supersensitive oxidation-responsive biodegradable PEG hydrogels for glucose-triggered insulin delivery. ACS Appl Mater Interfaces 2017;9:25905–14. 28714308 10.1021/acsami.7b08372

[28] Lin X , Konno T, Takai M, Ishihara K. Redox phospholipid polymer microparticles as doubly functional polymer support for immobilization of enzyme oxidase. Colloids Surf B Biointerfaces 2013;102:857–63. 23107964 10.1016/j.colsurfb.2012.09.024

[29] Volpatti LR , Facklam AL, Cortinas AB, Lu Y-C, Matranga MA, MacIsaac C, Hill MC, Langer R, Anderson DG. Microgel encapsulated nanoparticles for glucose-responsive insulin delivery. Biomaterials 2021;267:120458. 33197650 10.1016/j.biomaterials.2020.120458

[30] Volpatti LR , Burns DM, Basu A, Langer R, Anderson D. Engineered insulin-polycation complexes for glucose-responsive delivery with high insulin loading. J Control Release 2021;338:71–9. 34391834 10.1016/j.jconrel.2021.08.017

[31] Matsumoto A , Chen S. A boronate gel-based synthetic platform for closed-loop insulin delivery systems. Polym J 2021;53:1305–14.

[32] Yoshida K , Hasebe Y, Takahashi S, Sato K, Anzai J. Layer-by-layer deposited nano- and micro-assemblies for insulin delivery: a review. Mater Sci Eng C Mater Biol Appl 2014;34:384–92. 24268273 10.1016/j.msec.2013.09.045

[33] Lee J , Ko JH, Mansfield KM, Nauka PC, Bat E, Maynard HD. Glucose-Responsive trehalose hydrogel for insulin stabilization and delivery. Macromol Biosci 2018;18:e1700372. 29665232 10.1002/mabi.201700372 PMC5986559

[34] Liu X , Li C, Lv J, Huang F, An Y, Shi L, Rujiang M. Glucose and H_2_O_2_ dual-responsive polymeric micelles for the self-regulated release of insulin. ACS Appl Bio Mater 2020;3:1598–606. 10.1021/acsabm.9b01185 35021650

[35] Zhang S , Xin P, Ou Q, Hollett G, Zhipeng G, Wu J. Poly(ester amide)-based hybrid hydrogels for efficient transdermal insulin delivery. J Mater Chem B 2018;6:6723–30. 32254689 10.1039/c8tb01466c

